# Glutamine metabolic enzymes: to filament or not to filament?

**DOI:** 10.1042/BST20253136

**Published:** 2026-04-29

**Authors:** Raquel A.C. Machado, Ivan Rosa e Silva, Thaís Fontes-Milz, Yuan Chang-Halabi, Jhon A. Vargas, Andre L.B. Ambrosio, Sandra M.G. Dias

**Affiliations:** 1 Brazilian Biosciences National Laboratory, Brazilian Center for Research in Energy and Materials, 13083-970 Campinas, Brazil; 2 Laboratory of Nanophysiology and Structural Biology, Facultad de Ciencias Biológicas, Pontificia Universidad Católica de Chile, Av. Libertador Bernardo O'Higgins 340, Santiago 8331150, Chile; 3 Departamento de Química Orgánica, Escuela de Química, Facultad de Química y de Farmacia, Pontificia Universidad Católica de Chile, Santiago 7820436, Chile; 4 Sao Carlos Institute of Physics (IFSC), University of Sao Paulo (USP), Sao Carlos, Sao Paulo 13563-120, Brazil

**Keywords:** glutamine pathway, metabolic enzyme filamentation, supramolecular structure

## Abstract

The self-assembly of metabolic enzymes into filaments and other supramolecular structures is well-documented in bacteria and yeast but remains largely unexplored in mammalian cells. Enzyme filamentation is thought to play a crucial role in regulating metabolic networks by modulating enzymatic activity in response to cellular demands. Studies in yeast suggest that filament-forming enzymes are often positioned at key junctions of metabolic pathways, enabling dynamic activation or inactivation during growth or stress and directing metabolic flux accordingly. While this mechanism appears to be broadly conserved across species, the structural and functional characterization of human homologs of filamentous enzymes remains limited. In the present review, we focus on the glutamine metabolic pathway, highlighting enzymes known to form large self-assemblies in cells and examining the few cases where structural insights are available. Finally, we discuss the broader implications of metabolic enzyme filamentation in mammalian cells, underscoring its potential as an emerging area of research.

## Introduction

The ability of metabolic enzymes to form large self-assembling structures *in vitro* has been recognized for decades. However, only recently has the appreciation of membraneless, reversible subcellular condensates shed light on the critical role these structures play in regulating metabolic networks [1]. Numerous enzymes, spanning diverse biochemical compositions and biological pathways, have been found to assemble into nanoscale higher-order oligomers *in vitro* and/or to form self-assembled condensates within cells.

In yeast, a comprehensive screening identified 60 metabolic enzymes capable of organizing into distinct structures, classified as either foci or filament-like structures, as observed through fluorescence microscopy [2]. The study revealed that enzyme association frequently occurs at key junctions where major metabolic pathways connect with their branches, particularly under specific growth conditions. This observation suggests that these supramolecular structures may play a crucial role in promoting or inhibiting enzymatic activity in response to cellular demands, ensuring the proper direction of metabolic flux during periods of growth or stress.

Notably, this mechanism has been observed across various organisms, offering insights into how metabolic enzyme assembly has evolved to address distinct biological challenges. In some cases, there is strong evidence that the cellular foci and filament-like structures observed by fluorescence microscopy for metabolic enzymes under specific conditions are composed of polymeric filaments, as discussed throughout in the present review. In other cases, however, metabolic enzymes assemble into phase-separated condensates (e.g., glycolytic bodies and the purinosome) that do not involve the formation of polymeric filaments [3,4].

For example, cytidine triphosphate (CTP) synthase (CTPS), a rate-limiting enzyme responsible for catalyzing the final committed step in pyrimidine nucleotide biosynthesis, forms filaments called cytoophidia in bacteria, leading to the inhibition of its enzymatic activity [5]. CTP itself promotes filament formation, and the E277R interface mutation, which disrupts this assembly, has been shown to significantly impact cell growth and metabolism. In contrast, human CTPS filaments are induced by the presence of substrates (UTP and ATP), leading to enhanced enzymatic activity. Moreover, the filament architecture in human CTPS differs from that observed in bacterial CTPS, highlighting a distinct regulatory mechanism [6]. These findings suggest that the ability of metabolic enzymes to form higher-order assemblies represents an evolutionarily conserved regulatory strategy, although its functional consequences—activation or inhibition of enzymatic activity—have diverged across organisms. This mechanism is thought to ensure a fast, fine-tuned, and efficient adaptation to fluctuations in metabolic demand and/or response to environmental changes like pH [7], salinity [8], or temperature [9].

Besides CTPS, several fluorescently tagged yeast enzymes that transition from a diffuse to a spotted fluorescence intensity profile in response to metabolic state changes are also conserved in mammals. We have recently contributed to the work that described the polymerization of the human cystathionine beta-synthase [10]. This suggests that the ability to dynamically assemble into functional filament-like structures may be a broadly conserved regulatory mechanism in eukaryotic metabolism [2]. However, only a few studies have reported this phenomenon in mammalian cells, and even fewer have resolved the atomic structures of these higher-order assemblies, often using single-particle cryo-electron microscopy (cryo-EM) of purified recombinant proteins. Recent reviews provide detailed discussions on enzymes described to form filamentous structures *in vitro*, shedding light on their roles in enzyme regulation and metabolic control [1,11,12].

Interestingly, filamentation may also emerge in the context of pathological metabolic reprogramming, most notably in cancer [13]. A hallmark of cancer metabolism is the Warburg effect, where tumor cells favor glycolysis over oxidative phosphorylation, even in the presence of oxygen [14]. This shift is often accompanied by a strong dependency on specific nutrients like glutamine, driven by oncogene-regulated metabolic programs [15]. In this context, glutamine becomes essential for cell proliferation and immune modulation, making its metabolism a promising target for cancer therapy [16]. Glutamine can be metabolized by glutaminase 1 and 2 (GLS and GLS2) to produce l-glutamate, which is then converted into α-ketoglutarate—an essential intermediate in the tricarboxylic acid (TCA) cycle—by glutamate dehydrogenase (GDH) or aminotransferases [17]. Conversely, glutamine can be formed from glutamate and ammonia by the glutamine synthetase (GS) [18]. Glutamine also serves as the primary nitrogen source in proliferative cells, playing a crucial role in nucleotide biosynthesis and the production of non-essential amino acids, such as arginine, through asparagine synthetase (ASNS) [19].

**Figure 1:**
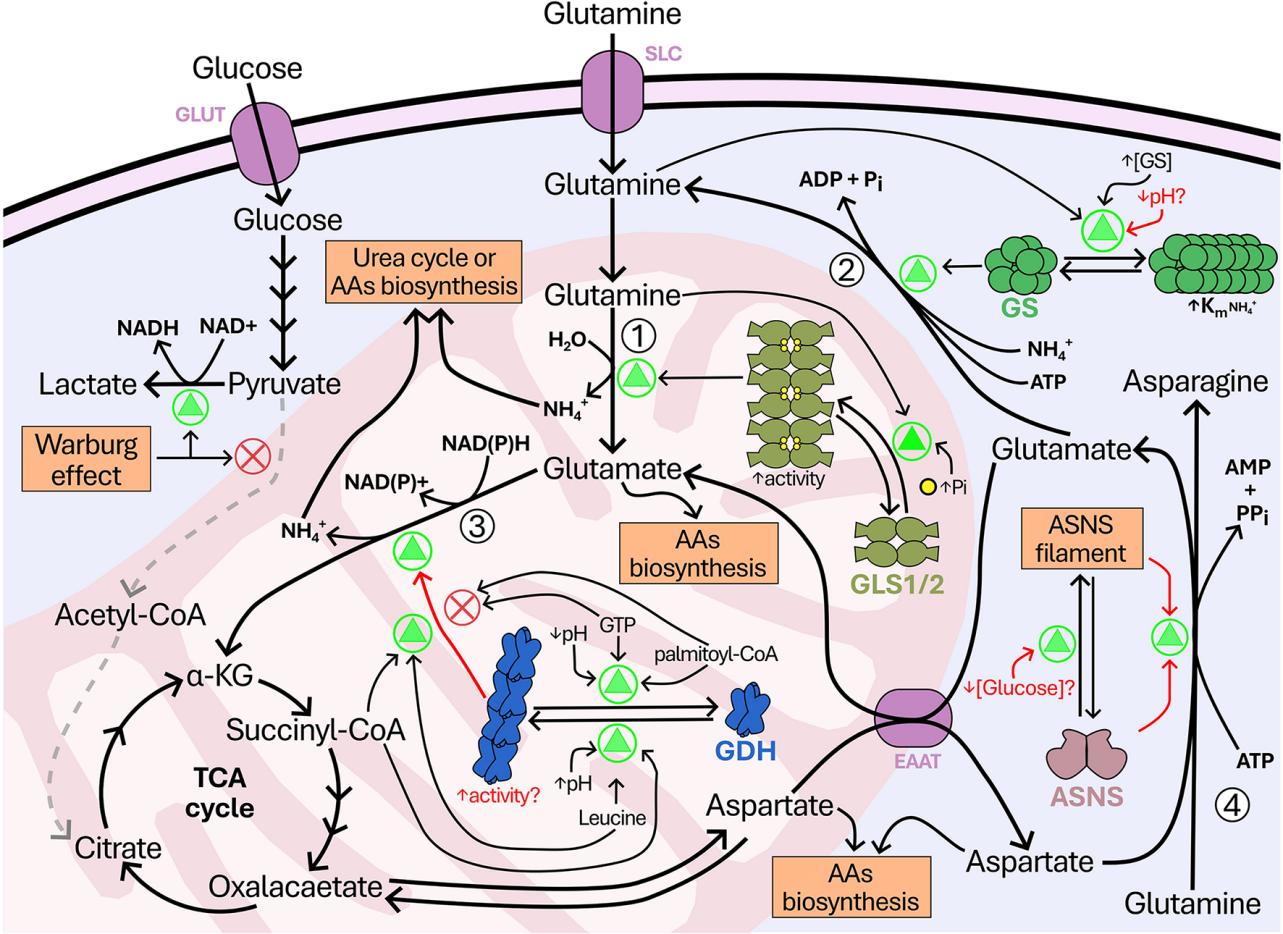
Schematic overview of glutamine and glucose metabolism highlighting enzyme filamentation and regulation Simplified representation of the metabolic crosstalk between glucose and glutamine metabolism in mammalian cells. Glucose is metabolized through glycolysis to pyruvate, which under the Warburg effect is primarily reduced to lactate. Glutamine enters the cell through specific transporters (purple) and fuels several pathways. Inside the mitochondria, glutaminase (GLS1/2) converts glutamine into glutamate (reaction 1). In the cytosol, GS catalyzes the ATP-dependent synthesis of glutamine from glutamate and ammonia, releasing pyrophosphate (PPi) (reaction 2). GS filaments are stabilized by glutamine and act as a feedback inhibition mechanism, decreasing enzymatic activity by lowering substrate affinity (increased Km). GLS1/2 filaments are stabilized by inorganic phosphate (Pi) binding, which enhances catalytic efficiency and promotes the active conformation of the enzyme. Glutamate is further oxidized by GDH to α-ketoglutarate (α-KG), replenishing the tricarboxylic acid (TCA) cycle (reaction 3). GDH filamentation is triggered by allosteric activators like leucine and succinyl-CoA, while inhibitors such as GTP and palmitoyl-CoA promote dissociation. However, the impact on enzyme activity and its high-resolution filament structure remain unclear. ASNS catalyzes the production of asparagine and glutamate from aspartate and glutamine (reaction 4); filament-like fluorescent signals of ASNS have been observed under nutrient stress, although its molecular structure remains unresolved. Green triangles (Δ) indicate reactions up-regulated or favored by specific metabolic or environmental cues (pH, phosphate, nutrient availability). Orange boxes highlight metabolic or structural phenomena, such as enzyme filamentation or metabolic reprogramming.

The ability of human and mouse GLS to form filaments both *in vitro* and *in situ* has been recently demonstrated [20]. In contrast, glutamine synthetase from humans (hGS) [21], yeast (Gln1p) [22], and bacteria (GlnA) [23]; yeast GDH (Gdh1p/Gdh2p) [2]; as well as yeast (Asn1p/Asn2p) [24] and human (hASNS) asparagine synthetase [25] have been shown to form filament-like structures *in situ* (and *in vitro*) under specific conditions (Table 1). Figure 1 represents an overview of glutamine metabolism-related mammalian enzyme filamentation and its regulation. Below, we summarize the current knowledge on the filamentation of these enzymes involved in the glutamine metabolic pathway across different organisms, examine the evolutionary conservation of the residues involved in the formation of these supramolecular structures, and discuss the potential molecular mechanisms underlying this process from an evolutionary perspective. We hope the present review will help researchers better understand enzyme filamentation and inspire further studies on its role in metabolic regulation and cancer.

**Table 1:** Glutamine pathway enzymes reported to form filament-like structure

Enzyme	Species investigated	*In situ* evidence for filamentation	Subcellular localization of the filament	Structural evidence for filamentation	Physical properties of the building block and the filament	Validated filament- regulating mutations	Regulatory mechanisms of filamentation	Functional implications of filamentation
Glutaminase	Human and mice (GLS)	Fluorescence microscopy and cryo-ET [20]	Mitochondria [20]	Cryo-ET of mammalian cell mitochondria (PDB: 8EC6 [20]) and cryo-EM structure of recombinant GLS (PDB: 8IMA [33])	Oligomeric building block [20]: P_i_-bound tetramer (dimer of dimers); Filament parameters [20]: Helical rise: ∼70 Å Helical twist: ∼48° Symmetry (cryo-EM data processing): D2	Constitutive-filament mutation: K320A, K320A + Y394A [13,34,76]; Filament-competent but catalytically defective mutations: Y466W [13], K320A + Y466W [76]; Filament-disrupting mutations: F355A^*^, F373A^*^, N375A^*^, F378A^*^, Q379A^*^ and Q416A^*^ ^*^tetramer-tetramer interface amino acid residues [13,76]	P_i_ *in vitro* and glutamine starvation *in situ* induces GLS filamentation [20]	GAC is active when assembled as a filament [13,20,33]; in cells, glutamine removal stabilizes GAC filaments impacting mitochondria morphology and survival [20]
	Human (GLS2)	Not reported	Not reported	Cryo-EM structure of recombinant GLS2 K253A (PDB: 8T0Z [13])	Oligomeric building block [13]: Tetramer (dimer of dimers); Filament parameters [13]: Helical rise: ∼66 Å Helical twist: ∼48° Symmetry (cryo-EM data processing): C1	Constitutive-filament mutation: K253A [13,77]	The GLS2 K253A mutant constitutively assembles active filaments *in vitro* in the presence of glutamine and in the absence of P_i_ [13]	GLS2 is active when assembled as a filament [13]
Glutamine synthetase	Bacteria (GlnA)	Not reported	Not reported	Cryo-EM structure of recombinant GlnA (PDB: 7W85 [23])	Oligomeric building block [23]: Dodecamer (dimer of hexamers in a back-to-back orientation); Filament parameters [23]: Helical rise: ∼97 Å Helical twist: ∼18° Symmetry (cryo-EM data processing): C6	Filament-disrupting mutations: H5A^*^ (mild), H13A^*^ (mild), H5A + H13A ^*^divalent metal ions coordination amino acid residues at the interface between two dodecamers [23]	GlnA filamentation is induced *in vitro* by divalent metal ions (nickel, cobalt, zinc) and dissociates upon substrate (glutamate/ATP) addition [23]	GlnA is inactive when assembled as a filament [23]
	Yeast (Gln1p)	Fluorescence microscopy and correlative light/electron microscopy [7]	Cytoplasm [7]	Crystal structure of recombinant Gln1p ΔN18 (PDB: 3FKY [22]	Oligomeric building block [22]: Decamer (dimer of pentamers in a back-to-back orientation - the active sites in the pentamer are related by a ∼30° rotation about the main axis of the oligomer); Higher order organization [22]: Crystallographic asymmetric unit: two adjacent decamers self-associate in a head-to-head orientation with the active sites of one pentamer shifted by ∼10° relative to those of the other pentamer	Constitutive-filament mutation: R23E, Y81A (mild) [7]; Filament-disrupting mutations: T49E, P83R and E186K [7]	Starvation induced by cell growing in phosphate buffer and acidic pH induces the formation of punctate and rod-like fluorescent Gln1p structures composed of filament segments approximately 1 μm in length in yeast cells [7]	Gln1p is inactive when assembled as a filament [7]
	Human (GS)	Not reported	Not reported	Cryo-EM structure of recombinant GS (PDB: 9OTQ, 9OTM, 9OTN, 9OTP [21])	Oligomeric building block [21]: Decamer (dimer of pentamers in a back-to-back orientation); Filament parameters [21]: Helical rise: ∼95 Å Helical twist: ∼26° Symmetry (cryo-EM data processing): D5	Filament-competent but catalytically defective mutations: R298A, E305A [21]; Filament-disrupting mutations: K52A, C53A (decamer-decamer interface amino acid residues) [21]	GS filaments are stabilized by glutamine [23]	GS filaments stabilized by glutamine show reduced catalytic efficiency
Glutamine dehydrogenase	Yeast (Gdh1p/2p/3p)	Fluorescence microscopy [2,43,45]	Cytoplasm [2,43,45]	Not reported	Not known	Not reported	*In situ*, glucose depletion induces the formation of filament-like structures, reversible with glucose replenishment [45]	Not known
	Bovine (GDH1/2)	Not reported	Not reported	Negative staining EM of bovine liver GDH1/2 [48]	Putative oligomeric building block: Hexamer (dimer of trimers); Filament parameters [48]: Number of linear chains: up to 4 tilted ∼28.5° relative to the main axis Helical rise: ∼800 Å	Not reported	*In vitro*, GDH1/2 filamentation is induced by alkaline pH and allosteric activators (leucine and succinyl-CoA), while acidic pH and inhibitors like GTP and palmitoyl-CoA, promote filament dissociation [49,50]	Not known
Aspargine synthetase	Yeast (Asn1p/2p)	Fluorescence microscopy [2,43,66,78]	Cytoplasm [2,43,66,78]	Not reported	Not known	Filament-competent but catalytically defective mutations: C1A [24]; Filament-disrupting mutations: A6E [72], R344A [24]	Glucose depletion induces Asn1p/2p filament formation, reversible with glucose replenishment [66]	Mutation A6E destabilizes ASNS protein levels, leading to fragmented filaments and impaired yeast growth [72]
	Human (ASNS)	Fluorescence microscopy [25]	Mitotic spindle [25]	Not reported	Not known	Not reported	Not known	Not known

## Glutaminase

GLS is a key enzyme involved in several critical metabolic pathways, facilitating the conversion of l-glutamine into ammonia and l-glutamate (Figure 1 and Table 2), which plays a crucial role in various biological processes, ranging from serving as a fuel in the tricarboxylic acid cycle to functioning as an excitatory neurotransmitter [26].

**Table 2:** Biochemical reactions of the glutamine metabolic enzymes

Glutaminase [79]	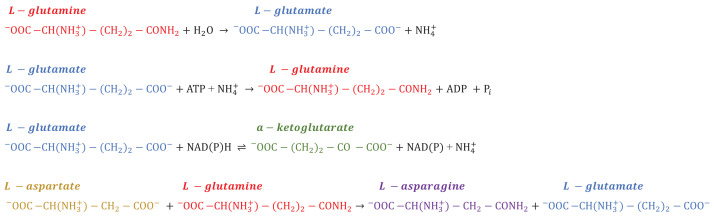
Glutamine synthetase [80]	
Glutamine dehydrogenase [81]	
Asparagine synthetase [82]	

In mammals, glutaminase is encoded by two genes, *GLS* and *GLS2. GLS* encodes the canonical splice variant, kidney-type glutaminase (KGA), as well as a C-terminally truncated isoform called glutaminase C (GAC), which lacks the ankyrin repeats (ANK) in the C-terminal region [27]. Meanwhile, *GLS2* encodes two more isoforms by alternative use of transcription initiation sites, the mitochondrial glutaminase B (GAB) and the shorter liver-type glutaminase (LGA) [28]. All glutaminase isoenzymes and isoforms present a conserved catalytic domain and N-terminus, while only KGA and GLS2 isoforms present the C-terminus ANK domain [27,29].

In 1970, Olsen et al. provided evidence that mammalian glutaminases purified from pig kidney exhibit different states of oligomerization, ranging from dimers and tetramers to long filamentous structures [30]. The authors showed that these structural transitions are induced by the presence of P_i_ and are associated with differential enzymatic activities. Notably, the “T-form,” corresponding to the dimeric state of glutaminases, was found to be practically inactive such that the addition of phosphate-borate promoted higher-ordered oligomerization to the active “P-B-form” [30].

**Figure 2:**
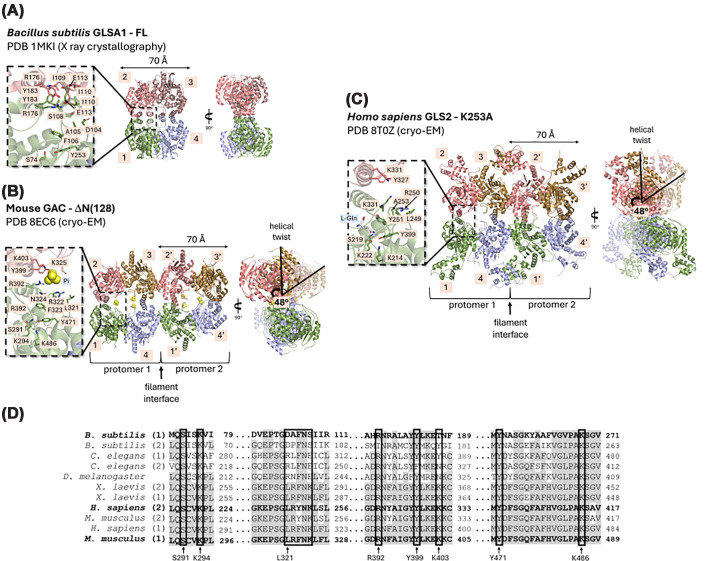
Structural basis for glutaminase filamentation (**A**) Cartoon representation of the full-length *Bacillus subtilis* glutaminase 1 (GLSA1) homotetramer obtained from the crystal structure deposited in PDB with accession code 1MKI (89). Active site amino acid residues are depicted as sticks. (**B**) Cartoon representation of the cryo-EM structure of inorganic phosphate-bound glutaminase C (GAC) core from *Mus musculus* (PDB: 8EC6 (17)), which assembles into a helical filament with a twist angle of 48°, as indicated. Active site amino acid residues are shown as stick representations. Inorganic phosphate molecules are shown in yellow. (**C**) Cartoon representation of the cryo-EM structure of l-glutamine-bound human liver-type glutaminase GLS2-K253A (PDB: 8T0Z (10)), which also assembles into a helical filament with a twist angle of 48°, as indicated. Active site residues as well as l-glutamine molecules are shown as sticks. (**D)** Amino acid sequence alignment of the N-terminal residues is shown for representative glutaminase sequences: *B. subtilis* (1) GLSA1 (UNIPROT ID O31465) e (2) GLSA2 (UNIPROT ID O07637); *Caenorhabditis elegans* (1) GLS1 (UNIPROT ID H2L2H3) and (2) GLS2 (UNIPROT ID Q19013); *Drosophila melanogaster* GLS (UNIPROT ID Q8IHB6), *Xenopus laevis* (1) GLSS (UNIPROT ID A0A8J0U0T6) and (2) GLSL (UNIPROT ID A0A8J0TWU6), *Homo sapiens* (1) GLSK (UNIPROT ID O94925) and (2) GLSL (UNIPROT ID Q9UI32); *M. musculus* (1) GLSK (UNIPROT ID D3Z7P3) and (2) GLSL (UNIPROT ID Q571F8). Conserved amino acid residues are depicted in gray.

In 2013, our group demonstrated that recombinant purified mouse GAC and KGA can be stabilized in filaments in the presence of P_i_ [31]. Additionally, we provided evidence that the stabilization capacity differs among GAC, KGA, and a GLS2 construct containing the common region shared by the LGA and GAB isoforms. GAC formed the longest and widest structures, followed by KGA, while GLS2 did not stabilize filaments in the presence of P_i_ [32]. More recently, we and others have uncovered the molecular mechanism underlying GLS and GLS2 activation by P_i_ and its role in filamentation [13,20,33] (Figure 2A–C). Binding of P_i_ at an allosteric dimer-dimer interface promotes a conformationally compact tetramer in which the four catalytic cores rotate inward, and the N-terminal domains expand outward, creating a competent filament interface [13,20,33] (Figure 2B,C). Thus, the activation of GAC and GLS2 is driven by P_i_-induced filamentation, which enhances their catalytic efficiency [13,20]. Cryo-EM structures revealed that P_i_ binds directly to residues such as hR317^GLS^/hR254^GLS2^ and hK320^GLS^/hK257^GLS2^ from the activation loop, promoting a structural shift that aligns hF318^GLS^/mY251^GLS2^ with hY466^GLS^/hY399^GLS2^ at the active site (Figure 2B,C) [13,20,33]. This interaction promotes a charge relay network between the catalytic triad (Figure 2B,C) (hS286^GLS^/hS223^GLS2^, hY466^GLS^/hY403^GLS2^, hK289^GLS^/hK226^GLS2^), activating the nucleophile required for glutamine hydrolysis.

While key conserved residues drive the filamentation of human and mouse GLS (GAC) at filament interfaces and allosteric regulation by P_i_, bacterial glutaminases feature a pre-stabilized allosteric site where P_i_ is not required for activation and lack a conserved filamentation interface [20] (Figure 2A,D). This structural organization restricts the flexibility of the activation loop and prevents filament formation, leading to distinct regulatory mechanisms for enzyme activity between bacterial and mammalian glutaminases. A phylogenetic analysis revealed that ANK repeats were acquired early in the evolution of glutaminases [27]. GAC, which does not have an ANK domain and exhibits enhanced self-assembly capacity, emerged later as a result of a retrotransposon event [20,27].

Successive stacking of tetramers drives the formation of mammalian GAC and GLS2 filaments, with each tetramer rotated by about 48° with respect to the adjacent one and a helical rise between 66 and 70 Å (Figure 2B,C). Filament formation occurs through a 6-α-helix interface on the lateral side of the catalytic domain near the lid loop that closes over the substrate-binding site, being mediated by hydrophobic and electrostatic interactions involving hF355^GLS^/hF288^GLS2^, hF373^GLS^/hF306^GLS2^, hF378^GLS^/hF311^GLS2^, hD412^GLS^/hD345^GLS2^, hQ416^GLS^/hQ349^GLS2^, stabilizing the enzyme in its catalytically active state (Figure 2B,C) [13,20,33]. This packing brings neighboring active sites into closer proximity than in isolated tetramers, effectively creating a row of aligned catalytic centers along the filament axis [13,20,33]. Besides, the GAC K320A [20] and GLS2 K253A [13] mutations cause the activation loop to adopt an active, Pi-bound-like conformation. Importantly, glutamine addition alone was sufficient to drive constitutively active forms of GLS2 K253A to form filaments.

Curiously, while P_i_ stabilizes GAC filaments [32], GLS2 filaments rapidly disassemble into tetramers once glutamine is fully converted to glutamate, demonstrating a direct link between filamentation and catalytic turnover [13]. In GLS2, ANK repeats further modulate activation by restricting conformational changes, which may explain its lower intrinsic activity and faster disassembly compared with GAC [13]. In this sense, the appearance of GAC suggests that its ability to form stable filaments represents a gain-of-function unique to this isoform. In line with this gain-of-function perspective, we demonstrated that GAC filaments bundle together inside elongated mitochondria under glutamine deprivation, preventing mitochondrial fission and resisting mitophagy, a phenomenon potentially uncoupled from glutamine metabolism [20].

Importantly, glutaminase filaments were observed in the hypoxic, nutrient-depleted core regions of pancreatic and gastric tumors, indicating a physiological relevance *in vivo* [34]. GLS filament formation serves as a dynamic structural switch that maximizes glutaminolysis, causing asparagine shortage, mitochondrial/ROS stress, and apoptosis [13,34]. Moreover, constitutively filamentous GLS mutants suppress tumor growth and xenograft formation, suggesting that promoting filamentation could represent a novel antitumor strategy in glutamine-addicted cancers [34].

## Glutamine synthetase

GS is a conserved enzyme found across a wide range of organisms, playing a crucial role in nitrogen metabolism [23]. This enzyme is responsible for the ATP-dependent conversion of l-glutamate and ammonium into l-glutamine (Figure 1 and Table 2) [18]. This process is essential for incorporating ammonia into cellular metabolism and for the biosynthesis of nitrogen-containing compounds [35].

GS is classified into three main types [36]: type I GS (GSI), encoded by *glnA*, predominantly found in prokaryotes and archaea, forming dodecamers (∼460 amino acids) [37,38]; type II GS (GSII), encoded by *glnII*, predominant in eukaryotes and some anaerobic bacteria, forming decamers (∼350 amino acids) [22,39,40]; and type III GS (GSIII), encoded by *glnN*, identified in some bacteria, forming dodecamers with more extended sequences (∼730 amino acids) [41].

**Figure 3:**
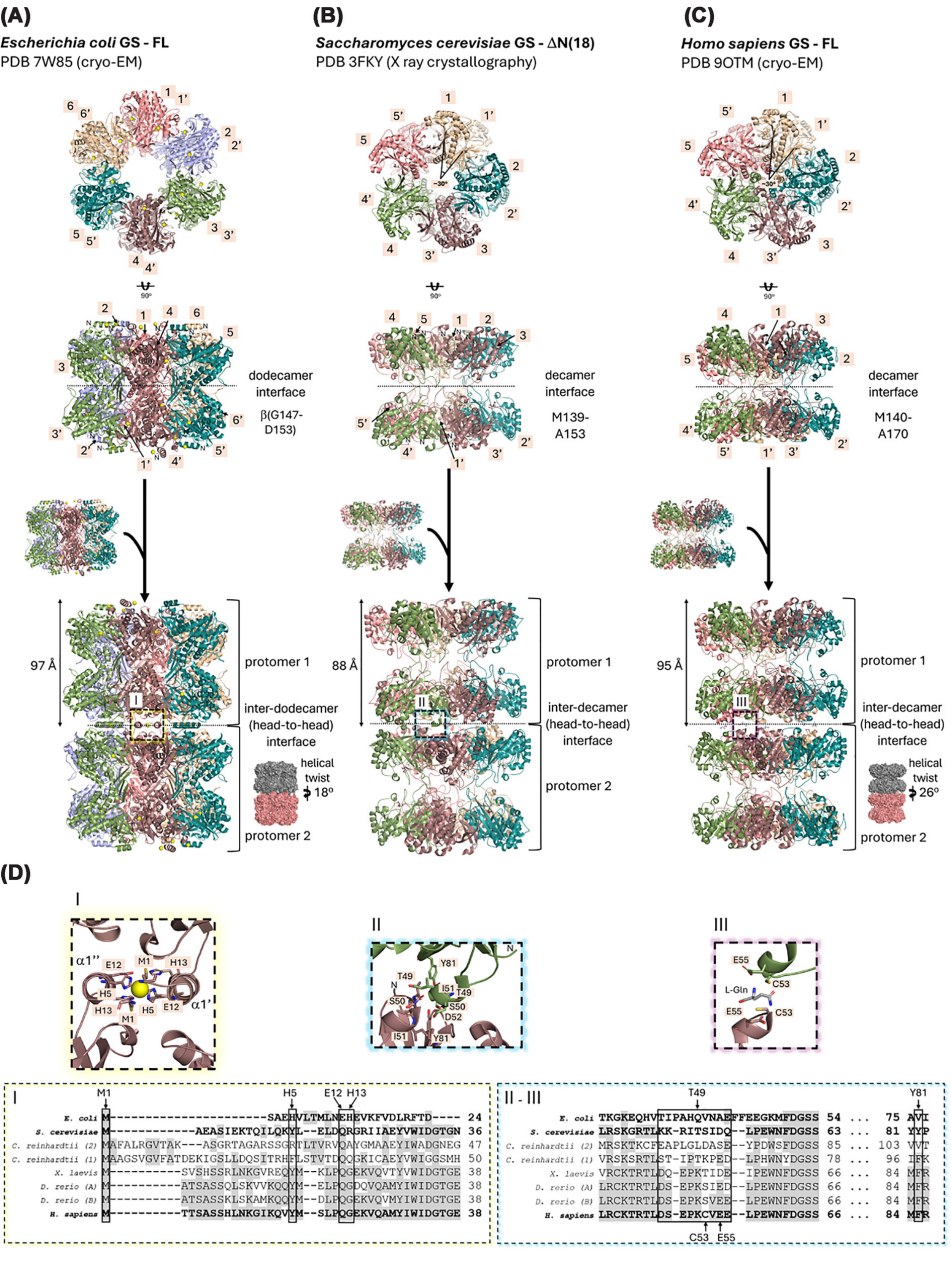
Structural basis for glutamine synthetase filamentation (**A**) Cartoon representation of the cryo-EM structure of the full-length (FL) *E. coli* GS (eGS) (PDB: 7W85 (20)). eGS assembles into a homododecamer through interactions between two hexamers and further filaments through head-to-head interactions mediated by N-terminal α-helix residues. Ni^2+^ ions are shown as yellow spheres. (**B**) Cartoon representation of the crystal structure of the N-terminally truncated (ΔN(18)) *Saccharomyces cerevisiae* Gln1p (sGS) (PDB: 3FKY (19)), forming a filament-like helical structure with a twist angle of ∼30°, as indicated. The decamer and the inter-decamer (head-to-head) interfaces are indicated. (**C**) Cartoon representation of the cryo-EM structure of the full-length (FL) *H. sapiens* GS (hGS) filament-like structure with a twist angle of ∼28°, as indicated, in the presence of l-glutamine (PDB: 90TM (18)). The decamer and the inter-decamer (head-to-head) interfaces are indicated. (**D**) Relevant amino acid residues in the inter-dodecameric interface of (I) eGS, and the inter-decameric interfaces of (II) sGS and (III) hGS, respectively, accompanied by excerpts of amino acid sequence alignments of representative glutamine synthetase sequences *i* (UNIPROT ID P0A9C5), *S. cerevisiae* (UNIPROT ID P32288), *Chlamydomonas reinhardtii* ((1)-UNIPROT ID Q42688 and (2)-UNIPROT ID Q42689), *X. laevis* (UNIPROT ID P51121), *Danio rerio* ((A)-UNIPROT ID Q7T2P7 and (B)-UNIPROT ID Q7ZVF2), and *H. sapiens* (UNIPROT ID P15104). Conserved amino acid residues are depicted in gray.

Structural studies using either X-ray crystallography or cryo-EM have provided a solid foundation for understanding GS oligomeric interfaces and catalytic mechanisms (Figure 3A–D). In particular, *Escherichia coli* GS (eGS) [23] self-organizes as a dodecamer arranged in two hexagonal rings stacked on each other (Figure 3A, PDB: 7W85 [23]), which is essential for catalysis. GlnA filaments adopt long, relatively rigid helical tube architectures (∼19 nm in diameter) assembled from laterally associated dodecamer stacks, characterized by a helical rise of approximately 97 Å and a helical twist of ∼18° per dodecamer [23]. EM data indicate limited bending over length scales of hundreds of nanometers [23]. The exposed N-terminal helix (α1) is critical for its filamentation through head-to-head inter-dodecameric interactions [23] (Figure 3A,D-I). *In vitro* assays revealed that the filamentation of purified dodecamers is induced by divalent metal ions such as Ni^2+^, through coordination with the residues H5 and H13 located in the first N-terminal helix (α1) [23] (Figure 3D-I). These histidine residues provide salt bridges and hydrogen bonds that stabilize the stacking of dodecamers (Figure 3D-I). Also, these filaments dissociate upon substrate (glutamate/ATP) addition, suggesting a reversible stress-response mechanism [23]. Interestingly, the methanogenic archaea *Methanosarcina mazei* requires the metabolite 2-oxoglutarate (2-OG) for inter-subunit stabilization of the dodecamer, directly coupling oligomerization to carbon availability [42].

Fluorescently tagged glutamine synthetase (Gln1p, sGS) from *S. cerevisiae* forms punctate and rod-like structures *in situ* under energy-depleted or low-pH conditions [7,43]. Fluorescent punctae represent clusters of aligned fiber segments (∼1 μm long), as shown by correlative light/electron microscopy [7,43]. Filament formation of sGS has been suggested to depend on two inter-ring interfaces: a decameric interface mediated mainly by hydrophobic interactions between the loop regions spanning M139-A153 (Figure 3B, PDB: 3FKY [22]) and a head-to-head interface primarily involving residues K45-D52 within the β-grasp domains of sGS (Figure 3D-II, PDB: 3FKY [22]). Although electron microscopy of purified heterologous sGS revealed abundant cylindrical particles with a head-to-head interface arrangement similar to that reported on the crystal structure of sGS [7], structural details remain limited, and the native interfaces have not been defined. Specific point mutations in sGS either hinder or facilitate filament formation. For example, mutations near or in the region of the head-to-head interface, including T49E, P83R, and E186K, abrogated the ability of sGS to filament in starved yeast while the mutant R23E had an increased propensity to assemble into filaments [7]. Specifically, R23E converts a conditional, pH-regulated inactivation mechanism into a constitutive filament state by making the interdecamer interface intrinsically strong, uncoupling filament formation from starvation signals [7].

Crystal structures of human GS (hGS), including N-terminal degron-truncated forms, show that the enzyme assembles into a dimer of pentamers, as also observed for the yeast glutamine synthetase, even in the presence of the sulfoximine inhibitor [44]. Cryo-EM studies further demonstrated that the decameric organization [40] is critical for catalysis (Figure 3C). In both humans and yeast, the N-terminal region is embedded within the structure, reducing charge exposure and potentially leading to more modulated interactions [22,40]. On the other hand, in eGS, the N-terminal region is exposed, facilitating electrostatic interactions [23]. Recent work by Greene et al. [21] showed that hGS forms helical filaments with a helical twist of 26° and a helical rise of ∼95 Å, stabilized by its product, glutamine, which binds at the inter-decamer (head-to-head) interface (Figure 3C,D-III, PDB: 90TM [21]). K52A and C53A mutations disrupt the decamer-decamer interface [21]. Filamentation in the presence of glutamine increases the flexibility of the well-conserved E305 active-site loop, reducing ammonia affinity and acting as a product-dependent feedback inhibition mechanism [21]. Thus, higher glutamine concentrations increase the fraction of GS in filamentous form, establishing a direct link between product levels and filament assembly [21].

## Glutamate dehydrogenase

GDH is an enzyme found across all living organisms, catalyzing the reversible conversion of l-glutamate into alpha-ketoglutarate using NAD(P)^+^ as a coenzyme, making it a key enzyme that interlinks carbon and nitrogen metabolism (Figure 1 and Table 2). In yeast, three cytosolic GDHs are present: Gdh1p and Gdh3p are NADP-GDHs involved in glutamate synthesis from ammonia and alpha-ketoglutarate, and Gdh2p works as a NAD-GDH, responsible for glutamate degradation, thereby producing ammonia and alpha-ketoglutarate [43,45,46].

Shen and colleagues demonstrated, *via* a screening of GFP-tagged GDH in budding yeast, that only Gdh2p forms fluorescent filament-like structures during the diauxic and stationary phases (post-glucose exhaustion) *in situ* [43]. Another study further revealed that up to 95% of yeast cells in the stationary quiescent phase displayed Gdh2p fluorescent filament-like structures, while Gdh1p fluorescent filament-like structures were observed in a smaller fraction (12%) of cells under the same condition [2]. For Gdh1p, filamentation is triggered by glucose depletion, but it is not induced by medium acidification, with these structures dissolving upon glucose replenishment [45].

In mammals, two primary GDHs are expressed, GLUD1 and GLUD2, both localized in the mitochondria. GLUD1 is involved in the synthesis and degradation of glutamate, playing a significant role in glutamate and nitrogen metabolism [46]. In contrast, GLUD2, found exclusively in humans and other apes, contributes to the recycling of glutamate during neurotransmission [46,47].

**Figure 4:**
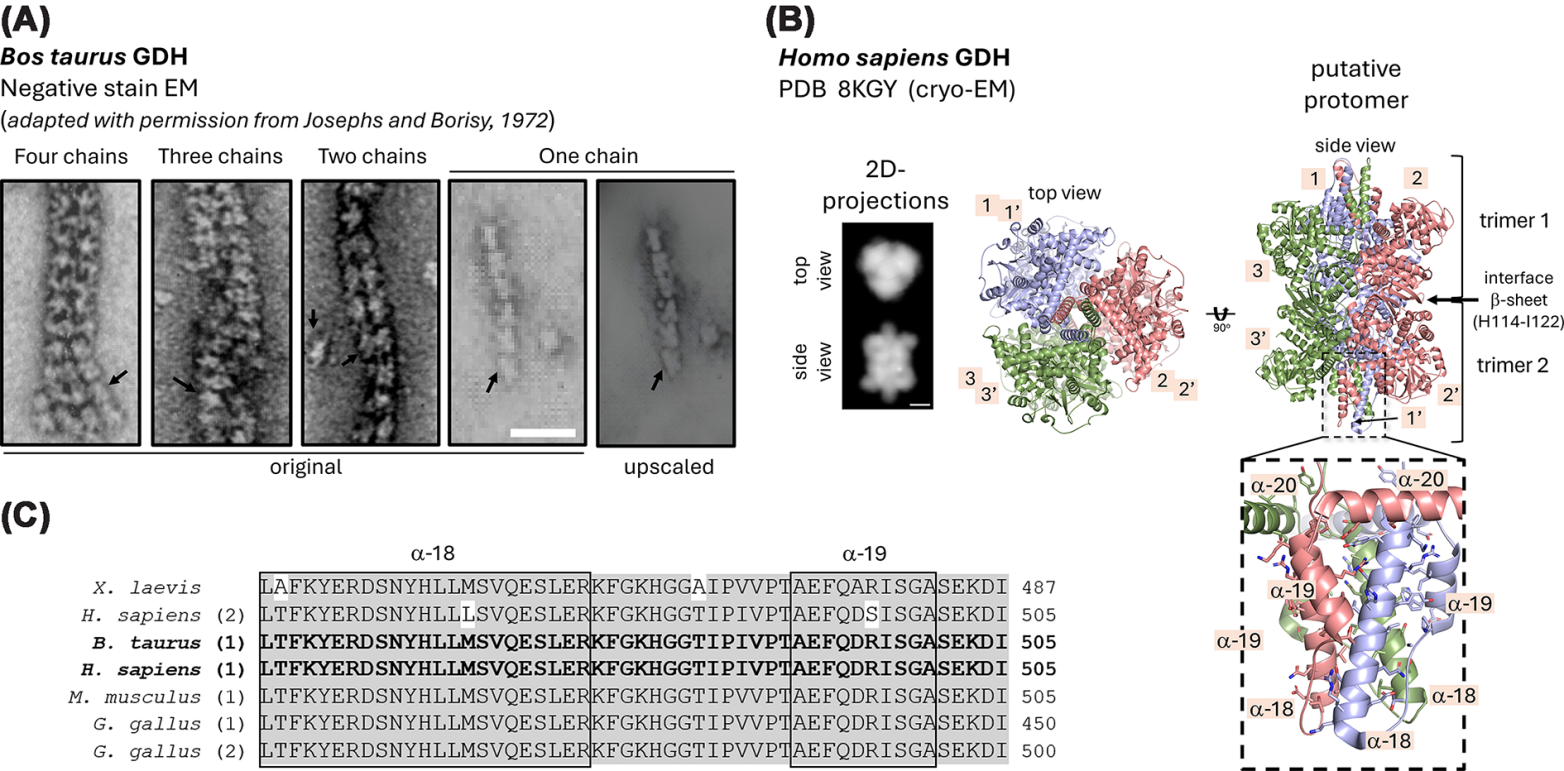
Structural insights into GDH filamentation (**A**) Negative stain EM micrographs of *Bos taurus* GDH showing filaments formed by different combinations of chains, as indicated. Adapted with permission from Josephs and Borisy (1972) (57). Structures resembling the putative protomer side views are indicated by arrows. Scale bar: 25 nm. (**B**) Cartoon representation of the cryo-EM structure of human GDH1 (PDB 8KGY (60)), composed of two homotrimers stacked to each other. Each trimer consists of three subunits (numbered 1–3 and 1′–3′). Key structural elements are highlighted. Low-resolution top and side-view projections of the cryo-EM structure, generated using ChimeraX (90), are also presented. Scale bar: 3 nm. (**C**) An amino acid sequence alignment of the N-terminal residues is shown for representative glutamine synthetase sequences: *X. laevis* (UNIPROT ID A0A974CEW5), *Gallus gallus* ((1) UNIPROT ID P00368 and (2) UNIPROT ID A0A1D5NT61), *M. musculus* ((1) UNIPROT ID P26443), *B. taurus* ((1) UNIPROT ID P00366), and *Homo sapiens* ((1) UNIPROT ID P00367 and (2) UNIPROT ID P49448). Conserved amino acid residues are indicated in gray. Amino acid residues critical for filamentation are indicated by boxes.

Purified GDH from bovine liver forms linear filaments *in vitro*, forming a helical tube composed of four linear chains, as observed by negative staining electron microscopy by Josephs and Borisy (1972) [48] (Figure 4A). Each linear polymer chain is oriented at an angle of around 28.5° relative to the filament axis, forming a helical pitch of approximately 800 Å [48]. Nonetheless, three alternative combinations of chain variants were also observed [48] (Figure 4A). As shown by Huang and Frieden (1962), the association of GDH filaments is promoted by alkaline pH, while acidic conditions favor filament dissociation [49]. Additionally, NADH and other effectors such as ADP stabilize the oligomeric state, highlighting a complex regulatory mechanism that integrates metabolic signals to control GDH filamentation. Allosteric activators, including leucine and succinyl-CoA, and inhibitors like GTP and palmitoyl-CoA, are reported to compete for the same binding site [50]. In general, activators promote bovine GDH self-assembly, while inhibitors induce the dissociation of these supramolecular structures.

The mammalian GDH putative protomer adopts a quaternary structure composed of two trimers stacked to each other (Figure 4B, PDB: 8KGY [51]) through their domain I regions [52–54]. In this orientation, β-strands of adjacent monomers form a 10-stranded β-sheet at the interface [52–54] (Figure 4B, PDB: 8KGY [51]). Hydrophobic interactions and hydrogen bonds between domain I residues of adjacent subunits anchor the assembly. This arrangement positions the catalytic domains at the interface between trimers, facilitating coordinated substrate binding and allosteric regulation [52–54]. The highly conserved C-terminal 48-residue helix–loop–helix motif (Figure 4B,C, PDB: 8KGY [51]) in animal GDH, called the antenna, extends from each subunit and wraps around adjacent subunits within the same trimer. Importantly, mammalian GDH is allosterically [55] by ADP and GTP through opposing allosteric effects on the catalytic cleft dynamics. ADP promotes catalytically competent open/relaxed states and prevents abortive complexes, whereas GTP, particularly with regulatory NADH, stabilizes a closed, inactive conformation [55]. Effector sites for GTP and NADH communicate with the antenna region, forming a network that directly links ligand binding to domain motions in the catalytic Rossmann fold [56,57].

Flexible hexamers could switch between active filamentous and dispersed states in response to cellular conditions. However, there is still no direct evidence for this hypothesis. Also, nuclear GDH binds actin/tropomyosin filaments [58,59], suggesting moonlighting roles in cytoskeletal stabilization. Despite subsequent reports on GDH filamentation *in vitro* and *in situ* and the high-resolution experimental structures of the *H. sapiens* [54,60,61] and *B. taurus* [62] GLUD1/2 homohexamers, the atomic structure of the GDH filament remains unavailable. Further studies are needed to determine the structural basis of GDH polymerization. Notably, a comparison between the low-resolution projections of the *B. taurus* GDH structure (Figure 4B) and the initial negative-stain electron micrographs reported by Josephs and Borisy (1972) [48] (Figure 4A) suggests that inter-chain side interactions play a crucial role in chain bundle formation, a phenomenon that warrants further investigation.

## Asparagine synthetase

ASNS catalyzes the synthesis of l-asparagine and l-glutamate from l-aspartate and l-glutamine (Figure 1 and Table 2) [63]. In bacteria, asparagine synthetase can exist in two forms, differentiated by their nitrogen sources: ammonia or glutamine [63]. In mammals, however, only one form is expressed, which uses glutamine as the nitrogen source. hASNS is classified as a class II glutamine amidotransferase or N-terminal nucleophile, as the hydrolysis of glutamine occurs in the N-terminal portion of the enzyme.

Yeast asparagine synthetase paralogs, Asn1p and Asn2p, are known to form cytoplasmic filament-like structures called cytoophidia at various growth phases in yeast cells [43]. ASNS cytoophidia display variable shapes (such as dots, short rods, and curved “snakes”) under the fluorescence microscope and frequently lie adjacent to or intertwined with CTPS cytoophidia, implying heterogeneous packing [64,65]. These structures are particularly observed during the diauxic phase, characterized by glucose depletion, and, more abundantly, in the stationary phase, when cells enter a quiescent state due to overall nutrient deprivation [2,24,43]. Filamentous fluorescence condensation also forms under glucose deprivation during the exponential phase and disassembles when glucose is reintroduced [66]. Interestingly, fluorescent-tagged Asn1p and Asn2p exhibit distinct filamentation capacities despite sharing 87.7% sequence identity, and colocalization analysis suggests the possibility of them forming a joint filament-like structure [24,66]. In this process, the Asn2p filament-like organization depends on Asn1p, while Asn1p can assemble independently. The mixed dependence of Asn1p and Asn2p on the formation of filament-like structures increases the likelihood of irregular bundle formation, complicating structural determination. To date, there is no high-resolution structure of yeast cytoophidia.

While Noree and colleagues [2] revealed that yeast asparagine synthetase forms a cytoplasmic filament-like fluorescent signal in response to nutritional stress, hASNS associates with centrosomes and mitotic spindles in dividing cells, suggesting a potential moonlighting role in mitosis, which awaits confirmation [25]. Interestingly, the introduction of a nuclear localization signal to the yeast wild-type protein or an activity disrupting mutation also promoted nuclear localization and mitotic spindle association in this organism, suggesting that its association with the mitotic spindle is independent of its enzymatic activity [67], which could represent a second case, along with the GAC glutaminase isoform, of gain-of-function filamentation.

**Figure 5:**
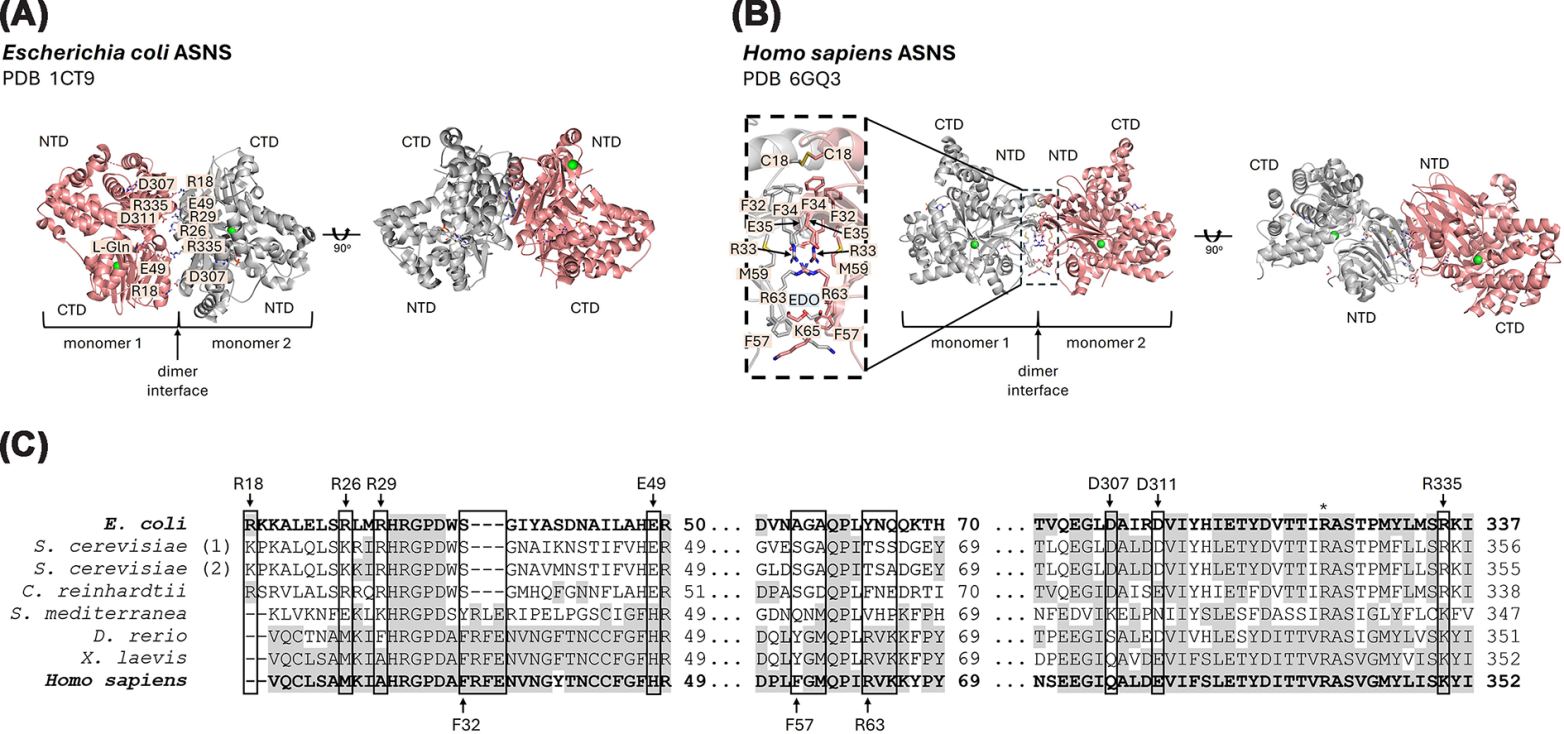
Structural basis for asparagine synthetase dimerization (**A**) Cartoon representation of the l-glutamine and AMP-bound AsnsB homodimer (PDB: 1CT9 (77)) depicting amino acid residues important for oligomerization. Ligands are shown as sticks. Chloride ions are depicted as green spheres. Dimer interface residues are shown as sticks. (**B**) Cartoon representation of the hASNS homodimer (PDB: 6GQ3 (79)), colored by residue conservation (cyan: low, violet: high), depicting amino acid residues important for oligomerization. 1,2-ethanediol (EDO) ligands are represented as sticks. Chloride ions are depicted as green spheres. The relevant amino acid residues in the dimer interface are highlighted in the insert. (**C**) Multiple amino acid sequence alignment of the relevant residues is shown for representative glutamine synthetase sequences: *E. coli* (UNIPROT ID P22106), *S. cerevisiae* ((1) UNIPROT ID P49089 and (2) UNIPROT ID P49090), *C. reinhardtii* (UNIPROT ID A8JG07), *Schmidtea mediterranea* (UNIPROT ID A0A1S6KMF3), *D. rerio* (UNIPROT ID A3KQK2), *X. laevis* (UNIPROT ID A0A974HF63), and *H. sapiens* (UNIPROT ID P08243). Conserved amino acid residues are indicated in gray. Amino acid residues critical for filamentation are indicated by boxes. Amino acid residue R344 (yeast sequence numbering) is indicated by an asterisk.

Bacterial ASNS, such as the *E. coli* AsnsA/B forms a functional homodimer, showing a head-to-tail organization that facilitates both l-aspartate and ATP substrate binding (Figure 5A, PDBs: 1CT9 and 12AS) [68,69]. In bacteria, a hydrophobic tunnel connects the N- and C-terminal domains, allowing efficient transfer of ammonia from the glutaminase site to the synthetase active site [69]. Unlike bacteria, hASNS adopts a head-to-head dimeric conformation in its active state because of intermolecular interactions involving amino acid residues 32-35 in the adjacent class II glutamine amidotransferase N-terminal domains and a disulfide bond (Figure 5B, PDBs: 8SUE, 9B6C, 6GQ3) [70,71].

Importantly, the polymerization of yeast Asn1p relies on its C-terminal synthetase domain, despite the current lack of structural evidence. The D330V mutation (equivalent to position D311 in AsnsA/B) enhances filamentation by altering homodimer interactions (Figure 5B,C). Since Asn2p relies on Asn1p for filament incorporation, this suggests a hierarchical assembly driven by heterodimer interfaces [24]. Also, the mutation A6E in the N-terminal region destabilizes Asn1p protein levels, leading to fragmented filaments and impaired yeast growth [72]. Furthermore, yeast Asn1p filamentation highly depends on amino acid residue R344 (equivalent to position R325 in eASNS, which is not present in the dimeric interface), indicating a direct link between enzymatic activity and filament assembly [24] that needs further clarification. However, there is currently no evidence of enzymatic inhibitors (such as acivicin, 6-diazo-5-oxo-l-norleucine, and azaserine) disrupting ASNS filamentation.

Although asparagine synthetase forms homodimers across domains of life (PDB IDs: 3RL6 [73], 1CT9 [69], 12AS [68], 4LNS [74], 8SUE [70], 9B6C [70], and 6GQ3 [71]), the resulting dimeric structures differ markedly, reflecting evolutionary divergence in catalytic mechanisms and substrate utilization. There is no evidence supporting the ASNS homodimer as the protomeric unit involved in filament formation. However, ASNS filament formation seems to be governed by structural elements, including dimerization interfaces and catalytic domains, with organism-specific adaptations influencing polymerization behavior and function.

Unlike other glutamine amidotransferases, including CTPS, for which pH-dependent filamentous cytoophidia and bundles with well-defined helical parameters (helical rise of 105 Å and helical twist of 40°) have been resolved by cryo-EM [75], no equivalent ASNS filament reconstructions are available. This is consistent with increased assembly flexibility, the presence of multiple assembly modes, or a lack of strict long-range helical symmetry. In yeast, Asn1p formation of fluorescent filament-like structures is robust only under specific stress conditions—such as carbon limitation, the diauxic shift, or stationary phase—and the condensates rapidly disassemble upon nutrient repletion, consistent with a reversible, stress-responsive state rather than a constitutive crystalline polymer. In humans, *ASNS* expression is tightly regulated at the transcriptional level by amino acid deprivation and ER stress; filament-like structure formation appears context-dependent and has not yet produced isolated, homogeneous filaments suitable for high-resolution in vitro cryo-EM analysis. Consequently, it remains unclear whether ASNS filament-like structures can adopt a single dominant helical architecture under well-defined biochemical conditions or whether they are intrinsically more flexible and multistate than CTPS filaments. Further structural studies are needed to elucidate the molecular mechanisms underlying the functional consequences of ASNS formation of filament-like structures, which could be validated through site-directed mutagenesis of amino acid residues that determine supramolecular organization.

## Perspectives

- Enzyme filamentation is recognized as a fundamental layer of metabolic regulation that complements post-translational modification and transcriptional control. Recent single-particle cryo-EM and cryo-ET advances have provided the basis for enzymatic assembly into higher-order structures in response to metabolic cues, influencing cellular organization, robustness, and disease.
- Filamentation spans diverse, enzyme-specific architectures with distinct regulatory outcomes. In glutamine metabolism, GLS and GS form helical filaments, GDH builds tubular assemblies, and ASNS forms stress-induced higher-order structures, supporting a spectrum model rather than a single structural solution.
- Key challenges include defining the molecular basis of filament architecture, understanding how assembly modulates catalysis *in vivo*, and distinguishing stable filaments from dynamic condensates. Integrating high-resolution, time-resolved, and *in situ* methods will be critical to link structure with function, metabolism, and disease and to harness filaments as metabolic switches and therapeutic targets.

## Data Availability

No new data were generated for this article.

## Open Access

Open access for this article is provided via our Subscribe to Open programme, made possible through the support of our subscribing institutions. Learn more here: https://portlandpress.com/pages/open_access_options_and_prices#conditional.

## Abbreviations

2-OG: 2-oxoglutarate
ADP: adenosine diphosphate
ANK: ankyrin repeats
Asn1p/Asn2p: yeast asparagine synthetase
ASNS: asparagine synthetase
ATP: adenosine triphosphate)
cryo-EM: cryo-electron microscopy
cryo-ET: cryo-electron tomography
CTP: cytidine triphosphate
CTPS: cytidine triphosphate synthase
eASNS: *Escherichia coli* asparagine synthetase
eGS: *Escherichia coli* glutamine synthetase
GAB: glutaminase B
GAC: glutaminase C
GDH: glutamate dehydrogenase
Gdh1p/Gdh2p: yeast glutamate dehydrogenase
Gln1p: yeast glutamine synthetase
GlnA: bacteria glutamine synthetase
GLS: glutaminase
GS: glutamine synthetase
GTP: guanosine triphosphate
KGA: kidney-type glutaminase
LGA: liver-type glutaminase
NADP: nicotinamide adenine dinucleotide phosphate)
Pi: inorganic phospate
sGS: *Saccharomyces cerevisiae* glutamine synthetase
TCA: tricarboxylic acid
UTP: uridine triphosphate
